# β-Thalassemia Intermedia: A Bird’s-Eye View

**DOI:** 10.4274/Tjh.2014.0032

**Published:** 2014-03-05

**Authors:** Anthony Haddad, Paul Tyan, Amr Radwan, Naji Mallat, Ali Taher

**Affiliations:** 1 American University of Beirut Medical Center, Department of Internal Medicine, Beirut, Lebanon; 2 American University of Beirut Faculty of Medicine, Department of Physiology, Beirut, Lebanon

**Keywords:** Thalassemia, Thalassemia intermedia, Iron chelation, Ineffective erythropoiesis, iron overload

## Abstract

Beta-thalassemia is due to a defect in the synthesis of the beta-globin chains, leading to alpha/beta imbalance, ineffective erythropoiesis, and chronic anemia. The spectrum of thalassemias is wide, with one end comprising thalassemia minor, which consists of a mild hypochromic microcytic anemia with no obvious clinical manifestations, while on the other end is thalassemia major, characterized by patients who present in their first years of life with profound anemia and regular transfusion requirements for survival. Along the spectrum lies thalassemia intermedia, a term developed to describe patients with manifestations that are neither mild enough nor severe enough to be classified in the spectrum’s extremes. Over the past decade, our understanding of β-thalassemia intermedia has increased tremendously with regards to molecular information as well as pathophysiology. It is now clear that β-thalassemia intermedia has a clinical presentation as well as complications associated with the disease that are different from those of β-thalassemia major. This review is designed to tackle issues related to β-thalassemia intermedia from the basic definition of the disease to paramedical issues, namely the quality of life in these patients. Genetics and pathophysiology are revisited, as well as the complications specific to this disease. These complications include effects on several organ systems, including the cardiovascular, hepatic, endocrine, renal, brain, and skeletal systems. Extramedullary hematopoiesis is also discussed in this article. Risk factors are highlighted and cutoffs are identified to minimize morbidities in β-thalassemia intermedia. Several treatment modalities are considered by shining a light on the pros and cons of each modality, as well as the role of special pharmacological agents in the progress of the disease and its morbidities. Finally, health-related quality of life is discussed in these patients with a direct comparison to the more severe β-thalassemia major.

## Abstract (tr)

Beta talasemi beta globin zincirlerinin sentezindeki defekt sonucu gelişen alfa/beta dengesizliği, inefektif eritropoez ve kronik aneminin sonucudur. Talasemi spektrumu geniş olup, bir uçta hiçbir klinik bulgusu olmayan bireyler varken, diğer uçta bulunan talasemi majorlu hastalar hayatın birinci yılında derin anemi ve hayatta kalabilmek için düzenli transfüzyon gereksinimi ile karakterize şekilde ağır seyirli olabilir. Bu iki ucun arasında ise talasemi major hastaları kadar ağır olmayan, ancak çok da hafif bir seyir göstermeyen hastaları tanımlamada talasemi intermedia terminolojisi kullanılmaktadır. Son on yılda β-talasemi intermedianın moleküler genetiği ve patofizyolojisi hakkındaki bilgilerimiz hızla artmıştır. β-talasemi intermedianın, β-talasemi majorden farklı, kendine özgü klinik başvuru özellikleri ve komplikasyonları olduğu netlik kazanmıştır. Bu derlemede β-talasemi intermedianın temel tanımlamalarından hastaların yaşam kalitesi gibi paramedikal konulara kadar meseleler ele alınmıştır. Genetiği, patofizyolojisi ve bu hastalığa özgü komplikasyonlarına değinilmiştir. Bu komplikasyonlar arasında kardiovasküler, hepatik, endokrin, renal, beyin ve kemiklerle ilgili olanlar vurgulanmıştır. Ekstramedüller hematopoez de bu makalede tartışılmıştır. Risk faktörleri özellikle vurgulanmış ve β-talasemi intermedialı hastalarda morbiditeleri minimuma indirebilmek için kritik eşik değerler belirtilmiştir. Değişik tedavi yaklaşımlarının iyi ve kötü taraflarına ışık tutulmaya çalışılmış ve bazı spesifik farmakolojik ajanların hastalığın progresyonu ve morbiditeleri üzerine etkisi tartışılmıştır. Son olarak bu hastalardaki sağlık ilişkili yaşam kalitesi, çok daha ağır seyirli β-talasemi majorlu hastalardaki ile karşılaştırılmıştır.

## INTRODUCTION

The first reports of thalassemic disorders date back to as early as 1925. The first cases were described in the pediatric population among subjects with anemia, peculiar facies, and other bony changes. The constellation of symptoms led to the hypothesis of a single disease entity back then [1]. Later on, the term “thalassemia” was coined by George Whipple [2]. Throughout the years, research focused on genetics and pathophysiology, and the theory of imbalance in globin chain production as a major culprit was revealed after Sir David Weatherall used labeled reticulocytes with radioactive amino acids to prove the defective production of alpha and beta chains [3,4,5]. Despite that theory being postulated a while ago, this pioneering work led to the modern definition of beta-thalassemia. It is nowadays considered as a defect in the synthesis of the beta-globin chains, leading to alpha/beta imbalance, ineffective erythropoiesis, and chronic anemia [6]. The diversity of the phenotypes in thalassemia make it diagnostically challenging. The spectrum of thalassemias is wide, with one end comprising thalassemia minor, which consists of mild hypochromic microcytic anemia with no obvious clinical manifestations, while on the other end thalassemia major is characterized by patients who present in their first years of life with profound anemia and regular transfusion requirements for survival [7].

In the middle lies thalassemia intermedia (TI), a term developed to describe patients with manifestations too mild to be considered thalassemia major and too severe to be called thalassemia minor. It was first used by Sturgeon, who suggested the term for those who fit into this category [8]. TI belongs to the non-transfusion-dependent thalassemia (NTDT) group, which also includes hemoglobin E/β-thalassemia and α-thalassemia intermedia (hemoglobin H disease). NTDTs extend from sub-Saharan Africa to the Mediterranean region and are also present in South and Southeast Asia. 

Despite a decreasing worldwide trend in the past few years, especially among the Mediterranean population, where prevalence and carrier rates were considerably high, thalassemias still remain a major public health burden [9].

**Genetics: Grasping the Concept **

Despite the considerable knowledge gained from research about TI in the past few years, diagnosis is still made on clinical grounds. The wide clinical spectrum of beta-thalassemia intermedia entails a wide range of presentations. Some patients remain asymptomatic for most of their lives with hemoglobin levels ranging between 7 and 10 g/dL, while others present during childhood and require transfusions for normal sustained growth (Table 1) [10].

One of the fundamental concepts of hematology is the fact that an equal number of alpha and beta chains should be present for proper hemoglobin physiology. While beta-thalassemia major arises from the total absence of the beta chains, TI arises from defective gene function leading to partial suppression of beta-globin protein production. It usually results from a homozygous or a compound heterozygous mutation [11]. In some instances, only one gene may be affected, making it dominantly inherited [12]. 

In most cases, the reason behind the milder phenotype of TI as compared to thalassemia major usually results from the interplay among 3 different mechanisms. The first is the inheritance of a mild or silent beta-chain mutation, which keeps a low level of beta chains, as opposed to its absence in more severe cases making less of an alpha/beta imbalance. The second is the inheritance of determinants associated with increased gamma chain production, which pair with unbound alpha chains. The third is the co-inheritance of alpha-thalassemia, which decreases the number of unpaired chains due to decreased alpha chain synthesis [13]. It is possible to inherit a triplicated or quadruplicated alpha genotype with beta heterozygosity and have a TI phenotype as a result. Many other factors come into play, with polymorphisms affecting bone, iron, and bilirubin metabolism affecting the disease. They are grouped under the umbrella of tertiary modifiers.

**Pathophysiology**

The hemoglobin molecule being usually composed of 2 alpha chains and 2 beta chains, any imbalance in the coupling of these fragments will naturally lead to an abnormal physiology, as equal alpha and beta chains need to be present for proper function. In beta-thalassemia in general, the absence or underproduction of the beta chain causes an imbalance with excess alpha chains deposited inside red blood cells. The latter process leads to oxidative damage to the membranes and eventually cell lysis [14].

This sequence of events is behind the concept of ineffective erythropoiesis. The resulting extra medullary hematopoiesis and marrow hypertrophy lead to expansion of the facial bones and obliteration of the maxillary sinuses, causing protrusion of the upper jaw and the peculiar thalassemic facies. It may also cause cortical thinning and pathologic fractures in long bones [15,16,17].

The resulting anemia is a consequence of ineffective erythropoiesis; however, hemolysis, in addition to being a contributor to the anemia, also has a role in other outcomes. Chronic anemia eventually leads to a state of continuous iron absorption, leading to iron overload and all of its resulting complications (cardiac, hepatic, endocrine, etc.). The triad of ineffective erythropoiesis, chronic anemia, and iron overload is at the heart of all morbidities related to TI. a contributor to the anemia, also vhas a role in other

**Complications**

**Figure 1:**
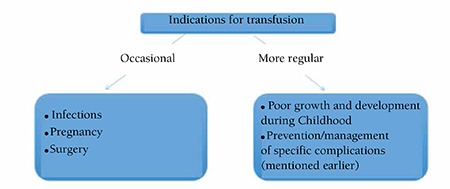
Indications for transfusion

When TI patients were compared to thalassemia major patients, many complications exclusive to TI suddenly surfaced. At the heart of these complications lies the triad of chronic anemia, ineffective erythropoiesis, and iron overload [18,19,20,21,22]. It is important to grasp a solid understanding of these complications in order to tailor management accordingly. A brief summary of the complications is provided in Figure 1, followed by specific complications and management in Table 2.

**Hepatobiliary Complications**

The accelerated hemolysis that occurs because of the instability in red blood cells leads to the formation of gallstones. For this reason, any symptomatic gallstone should be treated by a cholecystectomy. One additional consideration is the need to inspect the gallbladder in any patient who is undergoing splenectomy and consider intervention, since cholecystitis may be life-threatening in any splenectomized patient [23]. Since the majority of iron accumulation is in the liver in TI patients, there is an increased risk, mainly in non-chelated patients, of developing complications such as fibrosis, cirrhosis, and eventually hepatocellular carcinoma. This risk has been shown to be evident with higher iron loads and increased serum ferritin. Case reports from Lebanon and Italy have suggested a link between hepatocellular carcinoma and liver iron loading in hepatitis C-negative patients with TI [24,25].

**Extramedullary Hematopoiesis **

Extramedullary hematopoiesis is a physiological compensatory phenomenon occurring because of insufficient bone marrow function that becomes unable to meet circulatory demands [26]. Its occurrence in chronic hemolytic anemias remains highest, particularly in transfusion-independent TI [26,27]. Almost all body sites may be involved, including the spleen, liver, lymph nodes, thymus, heart, breasts, prostate, broad ligaments, kidneys, adrenal glands, pleura, retroperitoneal tissue, skin, peripheral and cranial nerves, and spinal canal [28,29,30,31,32]. These sites are thought to normally engage in active hematopoiesis in the fetus during gestation. The incidence of extramedullary hematopoiesis in patients with TI may reach up to 20% compared to polytransfused TM patients, for whom the incidence remains 1% [26,33,34]. A paraspinal location for the hematopoietic tissue occurs in 11%-15% of cases with extramedullary hematopoiesis [27,35]. Paraspinal extramedullary hematopoiesis mainly presents as pseudo-tumors, which may cause a variety of neurological symptoms due to spinal compression. However, it is thought that more than 80% of cases may remain asymptomatic, and the lesions are usually discovered incidentally by radiological techniques [36,37,38].

The male-to-female ratio reaches 5:1 [39]. Various clinical presentations have been reported, including back pain, lower extremity pain, paresthesia, abnormal proprioception, exaggerated or brisk deep tendon reflexes, Babinski response, Lasègue sign, paraparesis, paraplegia, ankle clonus, spastic gate, urgency of urination, and bowl incontinence [40].

**Leg Ulcers **

Leg ulcers are a common complication of TI, occurring in as many as one-third of patients with untreated or poorly controlled disease. They usually appear in the second decade of life and are generally located on the medial or lateral malleoli. The ulcers emerge after minor trauma and tend to expand rapidly [41]. They are slow to heal and tend to recur or become chronic, causing severe pain, disability, and esthetic problems that are difficult to manage for both patients and physicians [42].

The etiology of thalassemic leg ulcers seems to be multifactorial, with the main pathogenic mechanism appearing to be tissue hypoxia secondary to the anemia and the high affinity of fetal hemoglobin for oxygen [43].

**Others factors contributing to ulcer formation include:**

a) Abnormal rheological behavior of the diseased erythrocytes characterized by increased rigidity of their cellular membrane and enhanced adherence to endothelial cells,

b) Local edema due to venous stasis and possibly right heart insufficiency,

c) Repetitive local trauma and skin infections,

d) Hypercoagulability and prothrombotic tendency [44].

**A Hypercoagulable State **

The largest epidemiological study to date analyzed data from 8860 thalassemia patients (6670 with thalassemia major and 2190 with TI) and demonstrated that thromboembolic events (TEEs) occurred 4.38 times more frequently in TI than in thalassemia major patients [45]. The hypercoagulability in TI has been attributed to several factors, including a procoagulant activity of hemolyzed circulating red blood cells, increased platelet activation, coagulation factor defects, depletion of antithrombotic factors, and endothelial inflammation, among others [46]. These factors have been observed at a higher rate in splenectomized patients [46]. Clinical studies also confirmed that splenectomized TI patients have a higher incidence of TEE than non-splenectomized controls [45,47,48,49]. In the OPTIMAL CARE study, 73/325 (22.5%) splenectomized patients developed TEEs compared with 9/259 (3.5%) non-splenectomized patients (p<0.001) [47]. 

A study indicated that splenectomized TI patients who develop TEE are characterized by high nucleated red blood cell and platelet counts, and they are more likely to have evidence of pulmonary hypertension (PHT) and be transfusion-naive. Moreover, high nucleated red blood cell and platelet counts as well as transfusion naivety are associated with earlier development of TEEs following splenectomy [50].

**Silent Brain Infarcts**

Although strokes are uncommon in TI patients, one study showed that 37.5% of patients with TI have evidence of silent brain infarction on magnetic resonance imaging (MRI) [51]. More recently, in a study done on 30 splenectomized adults with TI, the rate of silent brain infarction was as high as 60% and it involved the subcortical white matter in all patients [52].

Recent studies have also documented a high prevalence of silent brain infarction, large cerebral vessel disease, and decreased neuronal function primarily in the temporal and parietal lobes in splenectomized adults with thalassemia intermedia [53]. There was a significant association between the occurrence of large-vessel cerebrovascular disease and high non-transferrin bound iron (NTBI) levels [54], and decreased neuronal function was observed more frequently in patients with a liver iron concentration (LIC) above 15 mg Fe/g DW [55].

**Pulmonary Hypertension**

Hemolysis is believed to play a key role in the development of PHT in TI patients. It has been shown that chronic hemolysis leads to nitric oxide depletion due to nitric oxide scavenging, arginine catabolism, and endogenous nitric oxide synthesis inhibition, as well as to enhanced platelet activation and increased endothelin-1 release [56,57]. All of those events in turn lead to a vasculopathy characterized by endothelial dysfunction, increased vascular tone, inflammation, hypercoagulability, and, finally, vascular remodeling and destruction of the pulmonary vasculature, which ultimately results in hemolytic anemia-associated PHT [56,58]. 

Autopsies of a large series of patients with TI revealed thrombotic lesions in the pulmonary arteries, which may have been due to circulating platelet aggregates [59]. Similar findings of multiple microthrombi, which were composed mainly of platelets, were seen in the pulmonary arterioles and microcirculation in autopsies of 2 splenectomized patients with thalassemia [60]. A high rate of PHT in splenectomized TI patients has been documented and attributed to a chronic thromboembolic state [61,62]. Moreover, elevated levels of circulating red blood cell-derived microparticles were detected in splenectomized patients with TI [63]. Whether they contribute to the development of PHT in this patient population warrants evaluation. A recent study by Derchi et al. established for the first time the presence of PHT by right heart catheterization in thalassemic patients. It also showed that the prevalence was higher in TI patients compared to those with thalassemia major [64]. 

**Renal Complications**

Different renal cell types have different resistances to the decrease of oxygen supply [65]. In vitro and animal models have shown that hypoxia may cause apoptosis of tubular and endothelial cells [66,67,68]. Other in vitro studies showed that hypoxia can induce a fibrogenic phenotype. A final result may be represented by activation of fibroblasts and accumulation of an extracellular matrix of resident renal cells [69,70,71,72]. Hence, it is apparent that chronic hypoxia causes proximal tubular cell dysfunction and interstitial fibrosis, which, in the presence of other renal risk factors, may lead to progressive renal disease.

Anemia causes renal defects through mechanisms different from those employed in hypoxia. Anemia affects the glomerulus by inducing renal hyper perfusion and glomerular hyper filtration [73,74,75]. The mechanism is thought to be a decrease in systemic vascular resistance and a subsequent increase in renal plasma flow [76]. This may represent a benefit in the short term [77]. However, over the long term, glomerular hyper perfusion may theoretically mediate progressive renal damage, as reported by experimental and clinical data [78]. Thus, it is theoretically possible that persistent anemia, such as that seen in thalassemia major patients, may contribute to a progressive decrease of the glomerular filtration rate [79].

Iron is a source of oxidative stress in biological systems. In thalassemic patients, the increased intracellular content of non-hemoglobin iron generates free oxygen radicals that bind to different membrane proteins, altering the morphology, function, and structure of membrane proteins [80]. Free iron can also directly catalyze lipid peroxidation by removing hydrogen atoms from the fatty acids that constitute the lipid bilayer of organelles [81].

**Iron Overload**

**Three main factors are responsible for the clinical sequelae of TI:** ineffective erythropoiesis, chronic anemia, and iron overload. Iron overload occurs primarily as a result of increased intestinal iron absorption but can also result from occasional transfusion therapy, which may be required to manage certain disease-related complications [18,19]. The pathophysiology of iron loading in TI appears similar to that observed in patients with hereditary forms of hemochromatosis [56] and is different from that seen in thalassemia major, where there is a predilection for NTBI accumulation. NTBI is a powerful catalyst for the formation of hydroxyl radicals from reduced forms of O2 [20]. Labile or “free” iron can convert relatively stable oxidants into powerful radicals. A recent cross-sectional study of 168 non-chelated patients with TI aimed to establish an association between LIC and a variety of serious morbidities noted in this patient population. Each 1 mg Fe/g DW increase in LIC was significantly associated with an increased risk of thrombosis, pulmonary hypertension, hypothyroidism, hypogonadism, and osteoporosis. LIC values of at least 6-7 mg Fe/g DW discriminated patients who developed morbidity from those who did not [21]. A more recent longitudinal follow-up over a 10-year period confirmed these findings in 52 non-chelated patients with TI, and a serum ferritin level of 800 ng/mL was the threshold above which all patients were at risk of developing morbidity [22].

**Management**

**General Considerations **

One way to approach management of TI is by dividing therapy into 3 broad categories. These include conventional modalities such as transfusion and iron chelation therapy, splenectomy, supportive therapies, and psychological support [82]. The non-conventional methods comprise gene therapy and fetal hemoglobin modulation, while stem cell transplantation remains the only curative and radical treatment. Quality of life in patients with TI has been a topic of interest lately, and it is clear that without any treatment, patients are at risk of experiencing more morbidities and poorer health-related quality of life [83,84,85]. Many of the standards of treatment nowadays are derived from years of experience and the evolving concept of evidence-based medicine. Despite many modalities being available, some of them remain experimental in nature or are at an early investigational stage. The most important ones are transfusion therapy, iron chelation, fetal hemoglobin induction, and stem cell transplantation. 

**Transfusion Therapy **

Although regular blood transfusions are the cornerstone of medical therapy in beta-thalassemia major, one of the most challenging therapeutic decisions in TI is when to initiate transfusion [86]. 

The main factors guiding the decision to transfuse are usually the development of signs and symptoms of anemia including growth failure and development failure. Despite a sporadic need for transfusions in many TI patients in situations such as infection, pregnancy, or surgery, patients should never be committed to a regular transfusion program unless the clinical picture dictates it. The main indications for a regular transfusion program remain growth failure, skeletal deformity, exercise intolerance, and declining hemoglobin levels because of progressive splenomegaly (Figure 1) [86,87,88].

The greatest impact of blood transfusions is in the pediatric group, where, in addition to reversing the anemia, it also carries the risk of alloimmunization. This phenomenon was found to be relatively common if a transfusion regimen was started after 12 months of age [89].

Some authorities recommend Rhesus and Kell phenotyping prior to transfusion [90], with the role of steroids for a short period (3-5 days) in preventing alloimmunization yet to be elucidated.

**Iron Chelation **

With transfusion therapy comes another very important consideration, that of iron overload. In addition to the natural iron-absorbing state of NTDT patients, the transfusional iron burden is also significant. In the OPTIMAL CARE study, patients who received both transfusions and chelation therapy had a lower incidence of complications than those who received transfusion alone or no treatment at all [47], hence showing the importance of appropriate therapy. The rate of iron loading in patients who do not receive transfusions is thought to be around 2-5 g/year [91], as opposed to 7.5-15.1 g/year in transfused patients [92]. The challenging matter in NTDT in general is the determination of the appropriate time for the initiation of chelation therapy. In 2008, a study by Taher et al. determined that serum ferritin in TI often underestimates the real iron burden. In comparison with MRI, ferritin was found to underestimate LIC [93]. 

Later findings emphasized the need for a reliable measure of body iron, and the MRI R2 and R2* showed a valid correlation between liver iron and body iron, becoming the preferred modality to guide therapy [93,94,95].

**Figure 2:**
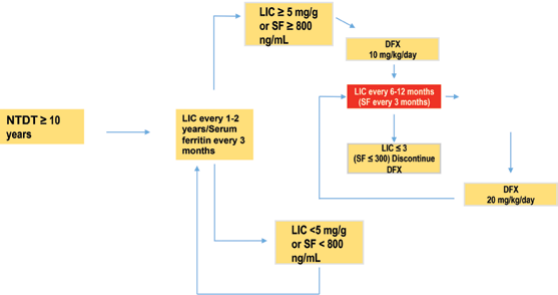
Algorithm for iron chelation Abbreviations: NTDT: non-transfusion dependent thalassemia, LIC: liver iron concentration, SF: serum ferritin, DFX: deferasirox.

Until recently, the cutoff for starting chelation was an LIC of 7 mg Fe/g DW or above. A recent study, however, by Musallam et al. showed that complications were more likely to occur at an LIC of 7 mg Fe/g DW, hence signaling the need to start chelating patients earlier [95]. The latest recommendations by the Thalassemia International Federation advocate LIC of 5 mg Fe/g DW and serum ferritin of 800 µg/L (Figure 2). Deferoxamine remains the gold standard in TI but the limitations of its use and its side effects have pushed pharmaceutical industries to look for other options. The main limitations reside in the fact that it needs to be administered either intravenously or subcutaneously, therefore increasing patient discomfort and negatively impacting quality of life [96,97]. Many studies evaluated deferoxamine in TI patients, and in 1988 Pippard and Weatherall concluded that the need for an oral chelator was very important despite deferoxamine delivering promising results in the studied group. Deferiprone (L1, Ferriprox) was the first oral chelator to be made available on the market. Small clinical trials demonstrated effective management of iron levels [98], but the lack of large-scale, placebo-controlled trials on deferiprone made the available data very limited. One of the recent products is deferasirox, an oral chelator with a once-daily dosage. It was developed to provide day-long chelation coverage with a suitable safety and efficacy profile [99,100]. The THALASSA trial was the first multicenter placebo-controlled double-blinded randomized trial to be conducted on TI patients, and it showed a sustained reduction in iron burden with deferasirox [101]. Further studies about its safety and efficacy are still ongoing. The latest new entry to the family of oral chelators was developed by Shire Pharmaceuticals, but it is still at an experimental stage with promising results on the horizon [102,103]. 

**HbF Inducers **

One postulated mechanism of action of hemoglobin F inducers is based on reducing the imbalance between alpha-globin chains and non-alpha chains [86]. These inducers may in fact increase the expression of gamma chain genes. The rationale behind inducing hemoglobin F is to decrease anemia symptoms and improve the clinical status of patients with TI [104]. 

In the Middle East, experience with HbF inducers is limited. Large series, however, come from Iran and India, where results have been promising, with some patients becoming completely transfusion-independent [105,106,107]. 

**Splenectomy**

All guidelines agree that physicians should adopt a guarded approach and restrict splenectomy to certain indications. Splenectomy should be avoided in children of <5 years of age because of a considerably greater risk of fulminant post-splenectomy sepsis (Table 3).

However, until a replacement for splenectomy is recommended through evidence-based guidelines, a large number of TI patients will continue to be splenectomized. These, alongside patients who had already undergone splenectomy, constitute a large proportion of TI patients at risk of TEEs [42]. Major adverse effects of splenectomy are sepsis, TEEs, thrombophilia, pulmonary hypertension, and iron overload. 

**Quality of Life**

Quality of life among patients with chronic diseases is of utter importance as there is a shift in medicine towards not only treating chronic diseases but also making sure that patients are well engaged and functional in their daily activities. A more specific term describing this state is health-related quality of life (HR-QoL). There is a scarcity of information in the literature when it comes to the evaluation of HR-QoL among β-thalassemia intermedia patients. A study by Musallam et al. compared HR-QoL in 32 adult β-thalassemia intermedia (non-transfused, non-chelated) and 48 β-thalassemia major patients [108]. The only significant difference between the groups was that patients with β-thalassemia major had a significantly longer median duration with a known thalassemia diagnosis while patients with β-thalassemia intermedia had a higher prevalence of multiple complications. This study suggested that a longer duration of known disease led to better adaptation and understanding of the disease’s nature; this could also explain how shorter duration of known disease causes high levels of stress and emotional anxiety [108]. A study that included pediatric patients also showed a higher percentage of children with β-thalassemia intermedia and impaired HR-QoL as compared to children with β-thalassemia major [109]. Another study that focused on patients’ mental health showed that a significant proportion of adult patients with both β-thalassemia major and intermedia showed evidence of depression (Beck Depression Inventory) and anxiety (State-Trait Anxiety Inventory) [109]. The risk of silent cerebral infarcts among these patients should not be excluded as an additional risk factor [110]. Nevertheless, more extensive large multicenter trials should be performed to further solidify our understanding of the patient’s quality of life, its implication on the course of the disease, and the possible ways to improve it.

## CONCLUSION

Although advances in TI are moving at a fast pace, many complications remain with no treatment guidelines. In addition, many of those complications can be prevented or adequately treated when caught at an early stage. This is why a low index of suspicion should be kept in mind. The only way to reduce the public health burden remains premarital screening and adequate genetic counseling. Even if birth rates have been declining in the world and the Middle East, adequate prevention strategies remain key. In addition, immigration patterns in the past few years are making thalassemia in general prevalent in many areas of the world. Novel therapeutic modalities are now emerging (JAK2 inhibitors, hepcidin modulators, apo-transferrin, etc.) and, in addition to the growing experience with hematopoietic stem cell transplants, the coming years may provide us with better outcomes. Most of these new modalities remain at an investigational stage at this point in time. Until they are finalized, it is fortunate that recent research in this field has helped elucidate a big part of what we did not know, therefore preventing fatalities.

## CONFLICT OF INTEREST STATEMENT

The authors of this paper have no conflicts of interest, including specific financial interests, relationships, and/ or affiliations relevant to the subject matter or materials included.

## Figures and Tables

**Table 1:**
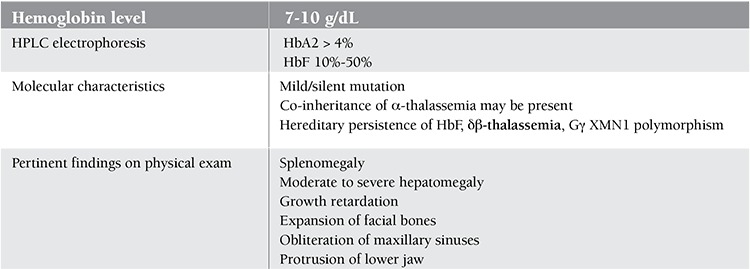
Characteristics and manifestations of β-thalassemia intermedia

**Table 2:**
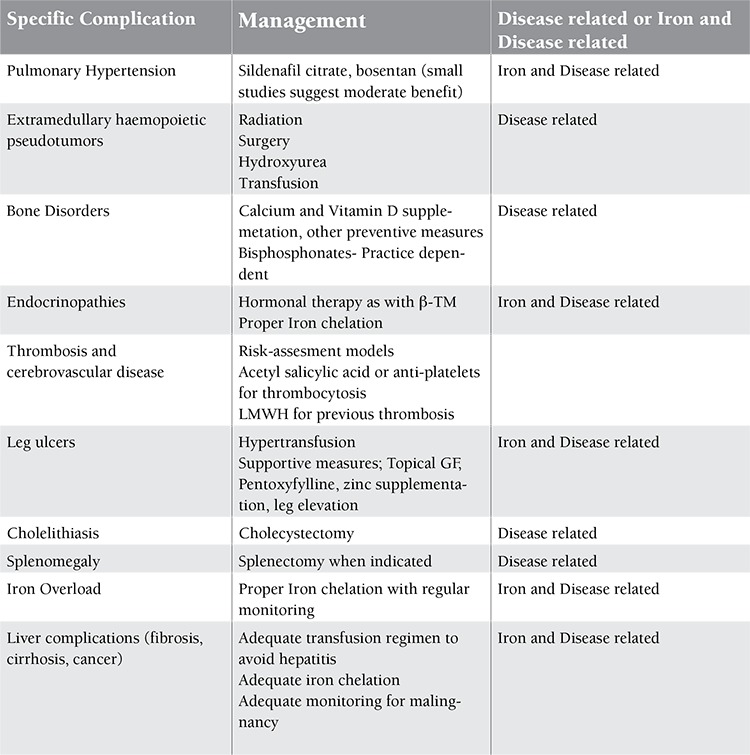
Specific complications of thalassemia intermedia and their management

**Table 3:**
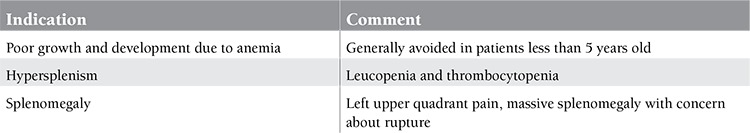
Indications for splenectomy
